# Rare variants in *KDR*, encoding VEGF Receptor 2, are associated with tetralogy of Fallot

**DOI:** 10.1038/s41436-021-01212-y

**Published:** 2021-06-10

**Authors:** Doris Škorić-Milosavljević, Najim Lahrouchi, Fernanda M. Bosada, Gregor Dombrowsky, Simon G. Williams, Robert Lesurf, Fleur V. Y. Tjong, Roddy Walsh, Ihssane El Bouchikhi, Jeroen Breckpot, Enrique Audain, Aho Ilgun, Leander Beekman, Ilham Ratbi, Alanna Strong, Maximilian Muenke, Solveig Heide, Alison M. Muir, Mariam Hababa, Laura Cross, Dihong Zhou, Tomi Pastinen, German Competence Network for Congenital Heart Defects, Marc-Phillip Hitz, Hashim Abdul-Khaliq, Felix Berger, Ingo Dähnert, Sven Dittrich, Anselm Uebing, Brigitte Stiller, Elaine Zackai, Samir Atmani, Karim Ouldim, Najlae Adadi, Katharina Steindl, Anita Rauch, David Brook, Anna Wilsdon, Irene Kuipers, Nico A. Blom, Barbara J. Mulder, Heather C. Mefford, Boris Keren, Pascal Joset, Paul Kruszka, Isabelle Thiffault, Sarah E. Sheppard, Amy Roberts, Elisabeth M. Lodder, Bernard D. Keavney, Sally-Ann B. Clur, Seema Mital, Marc-Philip Hitz, Vincent M. Christoffels, Alex V. Postma, Connie R. Bezzina

**Affiliations:** 1 Department of Clinical and Experimental Cardiology, Amsterdam University Medical Center, Amsterdam, The Netherlands; 2 Department of Medical Biology, Amsterdam University Medical Center, Amsterdam, The Netherlands; 3 Department of Congenital Heart Disease and Pediatric Cardiology, Universitätsklinikum Schleswig-Holstein Kiel, Kiel, Germany; 4 Division of Cardiovascular Sciences, School of Medical Sciences, Faculty of Biology, Medicine and Health, The University of Manchester, Manchester, UK; 5 Genetics and Genome Biology Program, The Hospital for Sick Children, Toronto ON, Canada; 6 Laboratory of Medical Genetics and Oncogenetics, HASSAN II University Hospital, Fez, Morocco; 7 Center for Human Genetics Leuven and Catholic University Leuven, Leuven, Belgium; 8 Centre de Recherche en Génomique des Pathologies Humaines (GENOPATH), Faculté de Médecine et de Pharmacie, Mohammed V University of Rabat, Rabat, Morocco; 9 Département de génétique médicale, Institut National d’Hygiène, Rabat, Morocco; 10 Division of Human Genetics, Children’s Hospital of Philadelphia, Philadelphia, PA USA; 11 Center for Applied Genomics, Children’s Hospital of Philadelphia, Philadelphia, PA USA; 12 Medical Genetics Branch, National Human Genome Research Institute, National Institutes of Health, Bethesda, MD USA; 13 Département de génétique, Hôpital Pitié-Salpêtrière, APHP Sorbonne Université, Paris, France; 14 Department of Pediatrics, Division of Genetic Medicine, University of Washington, Seattle, WA USA; 15 Division of Clinical Genetics, Children’s Mercy Hospital, Kansas City, MO USA; 16 Center for Pediatric Genomic Medicine, Children’s Mercy Hospital and School of Medicine, University of Missouri–Kansas City, Kansas City, MO USA; 17 HASSAN II University Hospital, Fez, Morocco; 18 University of Sidi Mohammed Ben Abdellah, Fez, Morocco; 19 Faculty of Medicine and Pharmacy, Medical Genetics and Oncogenetics Unit, Sidi Mohamed Ben Abdellah University, Fez, Morocco; 20 Institute of Medical Genetics, University of Zurich, Zurich, Switzerland; 21 University of Nottingham, Queen’s Medical Centre, Nottingham, UK; 22 Department of Pediatric Cardiology, Amsterdam University Medical Center, Amsterdam, The Netherlands; 23 Department of Pediatric Cardiology, Leiden University Medical Center, Leiden, The Netherlands; 24 Department of Cardiology, Boston Children’s Hospital, and Department of Pediatrics, Harvard Medical School, Boston MA, USA; 25 Department of Clinical Genetics, Amsterdam University Medical Center, Amsterdam, The Netherlands; 26 Manchester University NHS Foundation Trust, Manchester Academic Health Science Centre, Manchester, UK; 27 The Hospital for Sick Children, Toronto ON, Canada; 28 University of Toronto, Toronto ON, Canada; 29 DZHK (German Centre for Cardiovascular Research) Partner Site, Kiel, Germany; 30 Wellcome Sanger Institute, Wellcome Genome Campus, Hinxton, Cambridge, UK; 31 Universitätsklinikum Schleswig-Holstein Kiel, Kiel, Germany; 32 Universitätsklinikum des Saarlandes, Homburg, Germany; 33 Deutsches Herzzentrum Berlin, Berlin, Germany; 34 Herzzentrum Leipzig, Leipzig, Germany; 35 Friedrich-Alexander-Universität Erlangen-Nürnberg, Erlangen, Germany; 36 Universitätsklinikum Schleswig-Holstein, Kiel, Germany; 37 Universitäts-Herzzentrum Freiburg Bad Krozingen, Freiburg, Germany

## Abstract

**Purpose:**

Rare genetic variants in *KDR*, encoding the vascular endothelial growth factor receptor 2 (VEGFR2), have been reported in patients with tetralogy of Fallot (TOF). However, their role in disease causality and pathogenesis remains unclear.

**Methods:**

We conducted exome sequencing in a familial case of TOF and large-scale genetic studies, including burden testing, in >1,500 patients with TOF. We studied gene-targeted mice and conducted cell-based assays to explore the role of *KDR* genetic variation in the etiology of TOF.

**Results:**

Exome sequencing in a family with two siblings affected by TOF revealed biallelic missense variants in *KDR*. Studies in knock-in mice and in HEK 293T cells identified embryonic lethality for one variant when occurring in the homozygous state, and a significantly reduced VEGFR2 phosphorylation for both variants. Rare variant burden analysis conducted in a set of 1,569 patients of European descent with TOF identified a 46-fold enrichment of protein-truncating variants (PTVs) in TOF cases compared to controls (*P* = 7 × 10^-11^).

**Conclusion:**

Rare *KDR* variants, in particular PTVs, strongly associate with TOF, likely in the setting of different inheritance patterns. Supported by genetic and in vivo and in vitro functional analysis, we propose loss-of-function of VEGFR2 as one of the mechanisms involved in the pathogenesis of TOF.

## INTRODUCTION

Tetralogy of Fallot (TOF) is the most common form of cyanotic congenital heart defect (CHD).^1^ Although TOF can present in combination with extracardiac defects, in the majority of cases it presents as an isolated defect.^2^ An increased risk of CHD among first-degree relatives and offspring of TOF patients^3,4^ provides evidence for a genetic contribution to the disease etiology. A microdeletion on chromosome 22q11.2 is the most common genetic abnormality identified in patients with TOF, accounting for ~15% of cases.^5^ In addition, several other genes, mainly encoding cardiac transcription factors,^6,7^ have been implicated in TOF, although these account for a minority of patients, and the majority of cases remain genetically elusive. Over the last few years, dysregulated vascular endothelial growth factor (VEGF) signaling has been implicated in the pathogenesis of TOF. Exome sequencing studies have provided robust evidence that dominant, mainly truncating, pathogenic variants in *FLT4*, encoding VEGF receptor 3 (VEGFR3), are an important genetic cause of TOF.^8,9^ Furthermore, a candidate gene study^10^ identified rare variants in other VEGF signaling genes, including *KDR*, which encodes VEGFR2.^11^ Yet the causal role of rare variants in this gene has not been definitively established for CHD.

We conducted exome sequencing in a family with two children affected by TOF and identified biallelic missense variants in *KDR*. We subsequently conducted a large-scale genetic study in patients with TOF, including burden testing, and studies in gene-targeted mice and cell-based assays, to further explore the prevalence and nature of *KDR* genetic variation in patients with TOF and possible mechanisms leading to the etiology of TOF.

## MATERIALS AND METHODS

A detailed description of the methods is available in the Online Supplemental Methods.

### Genetic analysis and knock-in mouse model of index family with tetralogy of Fallot

We performed exome sequencing on DNA from two siblings of Moroccan descent affected by TOF with suspected recessive inheritance. Mouse lines harboring variants orthologous to the two *KDR* variants identified in the index family were generated by CRISPR/Cas9 targeting at Cyagen (Santa Clara, CA 95050-2709, USA).

### VEGFR2 phosphorylation assay

VEGFR2 phosphorylation status was assessed by western blot in yolk sac cells from knock-in mice and transfected HEK 293T cells. Values were compared to wild-type values by two-sided *t*-test per condition. A *p* value < 0.05 was considered significant.

### Additional patients with rare *KDR* variants

Patients with TOF were selected based on one of the following criteria, either (1) complex TOF, defined as TOF with absent pulmonary valve syndrome, pulmonary atresia, double outlet right ventricle, or with aortic arch abnormalities; or (2) TOF with a positive family history of CHD or (3) TOF patients of Moroccan descent. Patients from categories 1 and 2 were derived from the Dutch national biobank of adult patients with CHD (CONCOR) or recruited at the Amsterdam UMC (Netherlands) and at the Boston Children’s Hospital (USA). Patients from category 3 were recruited at the University Hospital of Fez (Morocco). *KDR* coding and copy-number variants were screened by Sanger sequencing and quantitative polymerase chain reaction (qPCR) respectively. In addition, we submitted *KDR* to the GeneMatcher database^12^ and considered both patients with CHD as well as patients with other phenotypes.

### Rare variant association analysis

We compared the burden of rare *KDR* variants in patients of European descent with (1) unselected TOF (primary analysis) or (2) patients with any type of CHD other than TOF (secondary analysis; these comprised a broad spectrum of CHDs, varying from mild to severe defects, described in detail elsewhere^9,13^) and controls. Cases were drawn from four different CHD cohorts for which exome or genome sequence data was previously generated.^8,9,13,14^ The non-Finnish European (NFE) subset of gnomAD v2 with exome data (gnomAD-NFE; *n* = 56,885)^15^ was used as control data set. We assessed rare variants in the canonical *KDR* transcript (ENST00000263923) in two different categories: protein-truncating variants (PTVs, i.e., nonsense, frameshift, and splice site variants) and missense variants. In addition, we analyzed the missense variants separately in five different domains of VEGFR2 (Supplementary table 1). Burden comparisons were performed using Fisher’s exact test. We applied a stringent Bonferroni corrected *p* value of < 0.007 (i.e., 0.05/7 tests) as significance threshold.

## RESULTS

### Familial tetralogy of Fallot with biallelic *KDR* variants

**Fig. 1:**
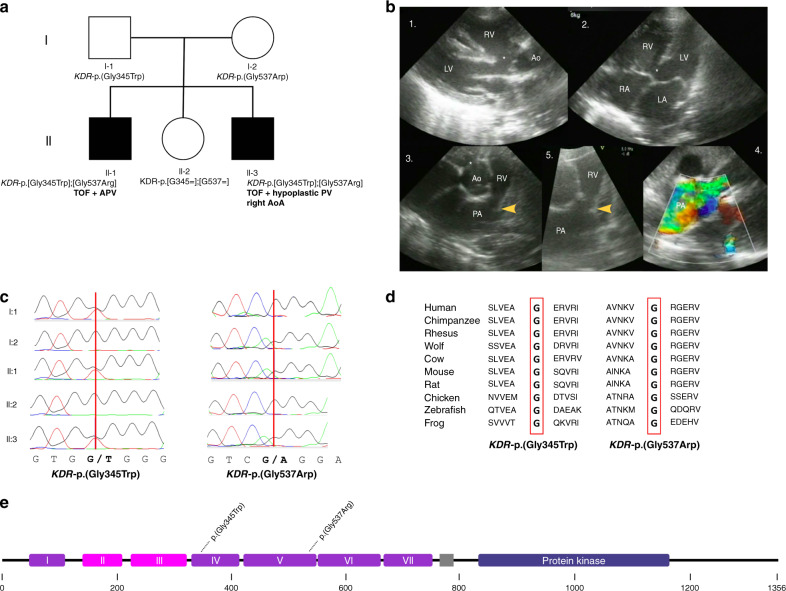
Identification of compound heterozygous *KDR* variants in a family with tetralogy of Fallot (TOF). (**a**) Pedigree of index family. The two affected children are marked with black symbols, the unaffected parents and sibling with white symbols. Genotypes are shown beneath. (**b**) Echocardiographic images of II-1 before cardiac surgery. 1: Parasternal long-axis view showing the large malalignment ventricular septal defect (VSD) (*) and the overriding of the aorta. 2: Four chamber view showing the VSD. 3: Short-axis view of the right ventricular outflow tract (RVOT), the dysplastic pulmonary valve (arrow) and dilated main pulmonary artery. 4: Color Doppler image showing turbulent flow (yellow-green) over the dysplastic valve consistent with a significant stenosis. 5: Detail of the RVOT, dysplastic pulmonary valve, and dilated PA. Note the dysplastic valve leaflets and small annulus. (**c**) Sanger sequencing chromatograms confirming a compound heterozygous inheritance in the two affected children. (**d**) Conservation of glycine residues at amino acid position 345 and 537 across species. (**e**) Location of *KDR*-p.(Gly345Trp) and *KDR*-p.(Gly537Arg) on the protein VEGFR2 subdomains are based on Roskoski.^20^ Ao aorta, AoA aortic arch, APV absent pulmonary valve, LA left atrium, LV left ventricle, PA pulmonary artery, PV pulmonary valve, RA right atrium, RV right ventricle.

The index family is a nonconsanguineous family of Moroccan descent with two children affected by a severe and complex form of TOF (Fig. 1a). Patient II-1 was diagnosed with a severe type of TOF, consisting of absent pulmonary valve syndrome and double outlet right ventricle (Fig. 1b). Patient II-3 was diagnosed with TOF with a severely hypoplastic pulmonary valve and a double outlet right ventricle, and a right-sided aortic arch. Detailed phenotypes are provided in Supplementary table 2.

There was no evidence of facial dysmorphisms, extracardiac manifestations, or neurodevelopmental delay in either child (current ages 17 and 7 years, respectively). Both parents (I-1 and I-2) and the unaffected sister (II-2) had normal echocardiograms and were healthy.

Exome sequencing, performed in II-1 and II-3, revealed 85 rare (minor allele frequency [MAF] < 0.1%) nonsynonymous or splice site variants that were shared among the two affected patients, none of which were homozygous. We excluded the presence of rare variants in a curated set of TOF genes^8^ (*n* = 77; Supplementary table 3). Because of a suspected recessive inheritance in this family, we prioritized biallelic variants. Two genes harbored multiple rare variants. After testing the variants in the parents, only one set of Sanger sequencing–validated compound heterozygous variants in *KDR* remained: *KDR*-p.(Gly345Trp) and *KDR*-p.(Gly537Arg) (Supplementary table 4). These variants were present in the two affected siblings in the compound heterozygous state, and were each inherited from an unaffected parent (Fig. 1c). The unaffected sister did not carry either *KDR* variant. *KDR*-p.(Gly345Trp) and *KDR*-p.(Gly537Arg) were not found in an internal data set of 390 controls of Moroccan descent or in any of the publicly available reference databases, including 3,065 individuals from North Africa and the Middle East in five population genetic data sets (Supplemental table 5). They were predicted to be deleterious by the in silico prediction tools SIFT and PolyPhen and had high CADD scores (35 and 34). The variants were located in the extracellular immunoglobulin(Ig)-like domains 4 and 5 of VEGFR2, and the affected residues were evolutionarily conserved (Fig. 1d, e).

The only other gene harboring multiple variants was *COL5A2*. Although these variants were shared by the affected patients, they were proven to be in *cis*, inherited from the unaffected father (Supplementary table 4).

### *Kdr*-p.(Gly535Arg) homozygous knock-in mice recapitulate the phenotype of *Kdr*^*-/-*^ mice

**Fig. 2:**
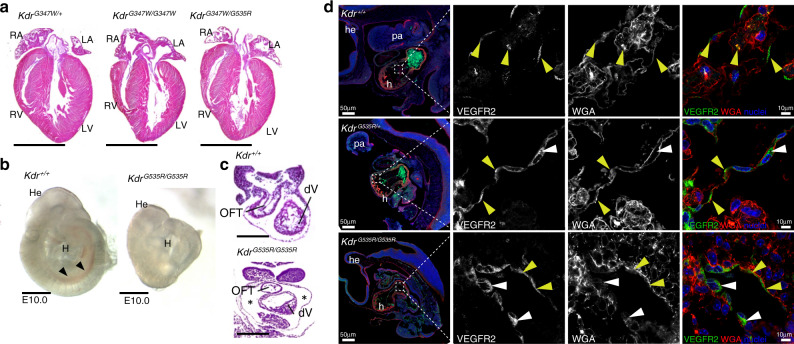
Characterization of orthologous genetic variants in mice. (**a**) Hemtoxylin and eosin (H&E) stained sections of *Kdr*^*G347W/+*^, *Kdr*^*G347W/G347W*^, and compound heterozygotes *Kdr*^*G347W/G537R*^ mice. Scale bar is 0.5 cm. (**b**) Whole mount images E10.0 *Kdr*^*+/+*^ and *Kdr*^*G535R/G535R*^ embryos. Normal vasculature (black arrows) as seen in *Kdr*^*+/+*^ are not visible in *Kdr*^*G535R/G535R*^ embryos, indicating impaired endothelial development. Scale bar is 1 mm. (**c**) H&E stained sections of the developing heart of the *Kdr*^*+/+*^ and *Kdr*^*G535R/G535R*^ embryos from (**b**), showing enlarged pericardial cavity in *Kdr*^*G535R/G535R*^ embryos (asterisk). Scale bar is 250 um. (**d**) Anti-VEGFR2 stained single optical sections of endocardial cells from *Kdr*^*+/+*^ and *Kdr*^*G535R/G535R*^ embryos. VEGFR2 is labeled green, WGA (cell membranes) is labeled red, and nuclei are labeled blue. Yellow arrowheads denote colocalization at the membrane of VEGFR2 and WGA and white arrowheads denote cytoplasmic VEGFR2. Scale bar is given in the figure. h heart, he head, pa pharyngeal arches, WGA wheat germ agglutinin.

Using CRISPR-Cas9 targeting, we generated knock-in mouse lines each carrying the mouse ortholog of the two variants found in the index family, i.e., *Kdr*-p.(Gly347Trp) (orthologous to *KDR-*p.[Gly345Trp]) and *Kdr*-p.(Gly535Arg) (orthologous to *KDR-*p.[Gly537Arg]), respectively. Heterozygous mice (*Kdr*^*G347W/+*^ and *Kdr*^*G535R/+*^) were born at normal Mendelian ratios and their hearts were morphologically indistinguishable from control littermates. This was also the case for compound heterozygous mice (*Kdr*^*G347W/G535R*^) and mice homozygous for the *Kdr*-p.(Gly347Trp) variant (*Kdr*^*G347W/G347W*^) (Fig. 2a and Supplementary table 6). However, we were unable to recover any mice homozygous for the *Kdr*-p.(Gly535Arg) variant (*Kdr*^*G535R/G535R*^) at birth (Supplementary table 7), suggesting that this variant may be embryonically lethal in the homozygous state. Previous studies have demonstrated that *Kdr* knockout animals die between embryonic day (E) 8.5 and E9.5 from severe vascular and hematopoietic abnormalities.^16^ Therefore, to determine whether *Kdr*^*G535R/G535R*^ mice mimic null animals, we examined *Kdr*^*G535R/G535R*^ embryos at E9.5. Although at this stage we found homozygous mice at normal Mendelian ratios (Supplementary table 7), homozygous mutants were smaller and paler than wild-type littermates with overall apparent necrosis and impaired endothelial development (Fig. 2b). Analysis of histological sections revealed an enlarged pericardial cavity in *Kdr*^*G535R/G535R*^ embryos (Fig. 2c). Overall, the phenotype of *Kdr*^*G535R/G535R*^ mice mimicked that of *Kdr* null mice,^16^ thus revealing that homozygosity of the *Kdr*-p.(Gly535Arg) variant has severe developmental consequences.

We next investigated the subcellular localization of VEGFR2 in endocardial tissue of E10.0 *Kdr*^*G535R/+*^ and *Kdr*^*G535R/G535R*^ mice. VEGFR2 signaling is highly active at this stage.^16^ Immunofluorescent staining of embryonic sections revealed VEGFR2 aggregation in the cytoplasm of endocardial cells in mutant embryos (*Kdr*^*G535R/+*^ and *Kdr*^*G535R/G535R*^) that was largely absent in wild-type littermates (Fig. 2d), where VEGFR2 predominantly localized to cell membrane.

### Familial *KDR* missense variants cause reduced VEGFR2 phosphorylation

**Fig. 3:**
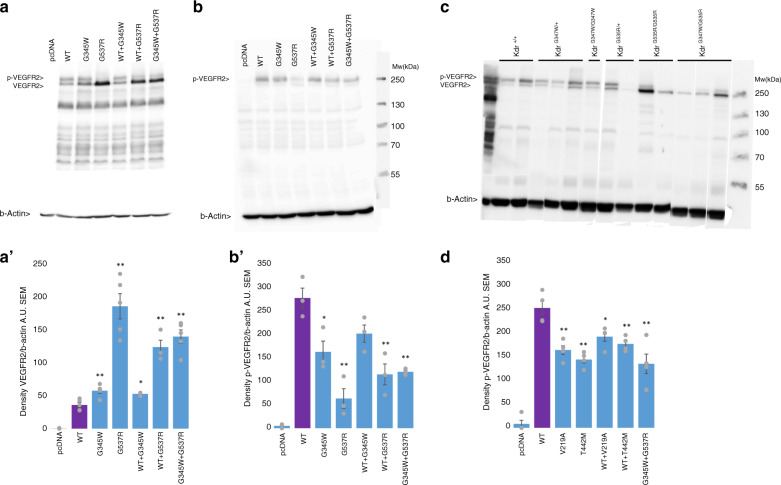
Phosphorylation assay. (**a**,**b**) Western blot analysis with VEGFR2 antibody or p-Y1175 antibody respectively in HEK 293T cells. The lanes contain (from left to right) HEK293T cells transfected with an empty vector (pcDNA), wild-type *KDR* (WT), *KDR-*p.(Gly345Trp) alone, *KDR-*p.(Gly537Arg) alone, *KDR-*p.(Gly345Trp) with WT, *KDR-*p.(Gly537Arg) with WT and *KDR-*p.(Gly345Trp) together with *KDR-*p.(Gly537Arg). (**a'**,**b’**) Quantification of western blots in (**a**) and (**b**). In (**a’**): *n* = 5 for each condition, except WT + *KDR-*p.(Gly345Trp) and WT + *KDR-*p.(Gly537Arg) (both *n* = 3). In (**b'**): *n* = 3 for each condition. *Standard two-sided *t*-test *p* < 0.05 compared to WT; **p < 0.01 compared to WT. (**c**) Western blot analysis with VEGFR2 antibody on yolk sac cells of wild-type and knock-in mice. Each lane represents one experiment. Gel has been edited to group genotypes; the unedited image can be found as Supplemental figure S1A. (**d**) Quantifications western blot analysis with p-Y1175 antibody in HEK 293T cells. The lanes contain (from left to right) HEK293T cells transfected with an empty vector (pcDNA), wild-type *KDR* (WT), *KDR-*p.(Val219Ala) alone, *KDR-*p.(Thr442Met) alone, *KDR-*p.(Val219Ala) with WT, *KDR-*p.(Thr442Met) with WT and *KDR-*p.(Gly345Trp) together with *KDR-*p.(Gly537Arg). *n* = 4 for each condition. *Standard two-sided *t*-test *p* < 0.05 compared to WT; **p < 0.01 compared to WT. Representative western blot can be found as Supplemental figure S1B.

In an effort to shed light on the biological mechanisms of the two *KDR* variants of the index family, we examined the phosphorylation of VEGFR2 at Tyr1175, one of its major phosphorylation sites^17^ and crucial for the recruitment of proteins in the signaling cascade downstream of VEGFR2.^17,18^ HEK 293T cells heterologously expressing the two identified variants or wild-type *KDR* (control) were stimulated with VEGF165. Western blot analysis on protein isolates using an antibody directed toward VEGFR2 showed two equally intense bands of the expected weight in cells expressing the wild-type VEGFR2. However, the lower band, representing unphosphorylated VEGFR2, was consistently and significantly more intense in cells expressing mutant VEGFR2, particularly those expressing the *KDR*-p.(Gly537Arg) variant and those co-expressing *KDR*-p.(Gly345Trp) and *KDR*-p.(Gly537Arg). The latter condition models the compound heterozygous state of the two affected patients from the index family (Fig. 3a). Western blot analysis with p-VEGFR2 antibody detected one major band at the expected weight (Fig. 3b), which had a significantly lower intensity for both variants expressed separately (40% and 80% reduction compared to wild-type for *KDR*-p.[Gly345Trp] and *KDR*-p.[Gly537Arg], respectively) and for the double mutant (60% reduction compared to wild type, *P* = 0.003; Fig. 3b). Similarly, western blot analysis of yolk sac cells from knock-in mouse embryos showed a seemingly modest reduction of p-VEGFR2 in *Kdr*^*G347W/G347W*^ mice, but a pronounced clear shift toward decreased relative phosphorylated VEGFR2 in *Kdr*^*G535R/+*^, *Kdr*^*G535R/G535R*^, and *Kdr*^*G347W/G535R*^ mice (Fig. 3c). Taken together, these data suggest that both variants cause decreased VEGFR2 phosphorylation at position Tyr1175. In line with the observed embryonic mortality and morphological phenotype of *Kdr*^*G535R/G535R*^ mice, decreased VEGFR2 phosphorylation at position Tyr1175 was most pronounced for *KDR*-p.(Gly537Arg) in HEK 293T cells and for the orthologous *Kdr*-p.(Gly535Arg) in mouse yolk sac cells.

Collectively, the genetic data from the family and the data obtained in mice and in vitro support a role for the two rare *KDR* missense variants in the index family, in which the functional effects of the *KDR*-p.(Gly537Arg) variant are more severe compared to those of *KDR*-p.(Gly345Trp). A summary of the characteristics of both variants is given in Supplementary table 8.

### *KDR* variants in different patient sets

**Fig. 4:**
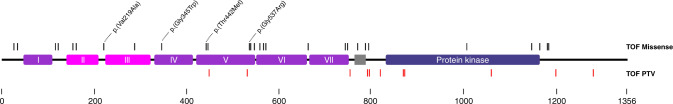
Schematic diagram of VEGFR2 with all variants identified in tetralogy of Fallot (TOF) patients. Location of rare *KDR* variants identified in patients with TOF (index family and 1,653 TOF probands). Variants are split into missense variants (black) and protein-truncating variants (PTVs) (red). Highlighted are the two variants identified in the index family, *KDR-*p.(Gly345Trp) and *KDR-*p.(Gly537Arg), and *KDR-*p.(Val219Ala) and *KDR-*p.(Thr442Met), that were functionally tested. Domains marked as I–VII represent the Ig-like domains 1 to 7. The transmembrane domain (gray box) separates the extracellular domain (left) of the intracellular domain (right). VEGFR2 subdomains are based on Roskoski^20^.

Based on the observation in the index family, we screened for coding variants and copy-number variants in *KDR*, using Sanger sequencing and qPCR respectively, in 82 patients with TOF who met at least one of the following criteria: (1) complex TOF, (2) positive family history of CHD, or (3) Moroccan descent. We identified a rare heterozygous missense variant (*KDR*-p.[Thr442Met], MAF gnomAD v2: 2 × 10^-5^) in one patient from the CONCOR biobank, with complex TOF, i.e., TOF and absent pulmonary valve syndrome (Supplementary table 9). Information about ethnicity was not collected, and unfortunately both the patient and her parents were unavailable for follow-up or genetic testing. Like the *KDR*-p.(Gly537Arg) variant from the index family, this variant was also located in Ig-like domain 5 (Fig. 4). Similar to the variants from the index family, western blot analysis with the p-VEGFR2 antibody demonstrated significantly reduced p-VEGFR2 for *KDR-*p.(Thr442Met) compared to wild type *KDR* (*p* < 0.001, Fig. 3d). No other coding variants or copy-number variants in *KDR* were detected in the remaining patients.

Through GeneMatcher we identified two more patients with complex TOF and a rare heterozygous variant in *KDR*, both PTVs: *KDR-*p.(R819*) and *KDR-*c.2614 + 1G>A (Fig. 4, Supplementary table 10). One of these was de novo, while the other PTV was inherited from the father who had three strokes stemming from an underlying autoimmune disease (suggested Takayasu syndrome). Moreover, these patients also had extracardiac abnormalities (Supplementary table 10).

In addition, we identified three patients with other types of CHD (with and without extracardiac abnormalities) and a heterozygous missense variant in *KDR* (de novo in two patients and inherited in the third), and two patients with a de novo KDR variant (one missense and one PTV, both heterozygous) had a noncardiac phenotype (Supplementary table 10). This suggests that rare *KDR* variants might also be associated with other phenotypes.

### Protein truncating variants are significantly enriched in patients with TOF

We next explored the causal role of rare *KDR* variants in TOF, by comparing the burden of rare variants in TOF patients with controls. A total of 1,569 patients with unselected TOF of European descent were drawn from different published^8,9,13,14^ and unpublished cohorts of patients with CHD (Supplementary table 11). Controls consisted of 56,885 individuals of non-Finnish European descent drawn from a reference population (gnomAD-NFE v2^15^). Rare *KDR* variants with a population maximum filtering allele frequency (FAF) < 0.01% (gnomAD v3^15^) were considered. As expected, we saw no difference in the frequency of synonymous variants between cases and controls (Supplementary table 12). We identified a total of 36 protein altering variants, 9 PTVs, and 27 missense variants (Fig. 4 and Supplementary table 9), in 35 patients with TOF. One patient had two different *KDR* missense variants (*KDR-*p.[Gly23Ser] and *KDR*-p.[Thr446Met]), but we could not assess compound heterozygosity due to lack of parental samples. The remaining 34 patients carried heterozygous *KDR* variants. In total, we identified rare KDR variants in 2.3% of patients with TOF. Parental samples were available for 8 of 35 patients and showed all 8 variants were inherited. There was a significant excess (46-fold enrichment) of PTVs in TOF cases compared to gnomAD-NFE controls (9 PTVs in 1,569 TOF cases versus 7 PTVs in 56,699 gnomAD-NFE controls; *P* = 7 × 10^-11^, Table 1). On the other hand, we detected no statistical enrichment of rare missense variants in patients with TOF compared to gnomAD-NFE controls (Table 1). Restricting the analysis to missense variants with a high in silico deleteriousness score (CADD > 20) or to ultrarare variants (FAF < 0.001%) did not influence the result. Because VEGFR2 is composed of different domains (Fig. 4 and Supplementary table 1),^19,20^ we next considered these distinct VEGFR2 subdomains separately in burden testing (Table 1). We observed a higher, though not statistically significant, burden of rare missense variants in the extracellular domain in patients with TOF compared to controls (19 variants in 1,569 TOF cases vs. 452 variants in 56,687 gnomAD-NFE controls; *P* = 0.08, Table 1 and Supplementary table 12). This signal seemed to be driven by variants in the ligand binding domain (7 variants in 1,569 TOF cases vs. 116 variants in 56,791 gnomAD-NFE controls, *P* = 0.04, Table 1), with two variants found in multiple patients (*KDR*-p.[Val159Met] in two patients and *KDR*-p.[Val219Ala] in three patients). Western blot analysis with the p-VEGFR2 antibody demonstrated a significantly reduced p-VEGFR2 for *KDR-*p.(Val219Ala) compared to wild-type KDR (*p* < 0.004, Fig. 3d); *KDR*-p.(Val159Met) was not tested.

**Table 1:** Results burden test in patients with tetralogy of Fallot (TOF) (FAF < 0.01%).

Variant type	Protein domain	Cases: *N* variants/total *N*	Controls: *N* variants/total *N*	Fold enrichment	*P* value
PTV	All	9/1,569	7/56,728	46	**7** **×** **10**^**-11**^
Missense	All	27/1,569	791/56,721	1.2	0.28
	All extracellular	19/1,569	452/56,687	1.5	0.08
	Ligand binding	7/1,569	116/56,791	2.2	0.049
	Ig-like 4-7	8/1,569	256/56,711	1.1	0.70
	Protein kinase	3/1,569	122/56,722	0.9	1
	All intracellular	8/1,569	339/56,722	0.9	0.87

Statistically significant *p* values after correction for multiple testing are highlighted in bold (Fisher’s exact test *p* < 0.007).

*FAF* filtering allele frequency, *PTV* protein-truncating variant.

Conversely, we did not identify any PTVs in patients with other types of CHD, neither did we observe an enrichment of missense variants in this group of patients in any of the subdomains (*n* = 2,312, Supplementary table 12).

## DISCUSSION

We identified compound heterozygous rare missense variants in *KDR* in a family with two siblings affected by a severe, complex form of TOF. Subsequent studies conducted in knock-in mice and cell-based assays showed significant diminished VEGFR2 autophosphorylation. In addition, the higher burden of PTVs in the 1,569 patients with TOF compared to controls further supports a loss-of-function mechanism. In total, 0.6% of patients with TOF in this study carried a PTV. The suggestive enrichment of rare missense variants in the extracellular domain of VEGFR2 in patients with TOF, together with the significant reduction in VEGFR2 phosphorylation we showed for multiple missense variants from this domain, suggests that disruption of residues within the extracellular domain of VEGFR2 might play a role in the pathogenesis of TOF.

The patients from the index family in this study carried biallelic variants in *KDR*. The observations made in knock-in mice and in heterologous studies in vitro support a causal role for both missense variants and may suggest a recessive inheritance model. Although we identified one additional patient with two different rare *KDR* missense variants, compound heterozygosity could not be confirmed in this patient due to lack of parental samples. *KDR* variants in all other patients in this study, as well as in a previously published study,^10^ were heterozygous. In some of these patients, the de novo occurrence of these variants provides support for dominant inheritance. On the other hand, the observation that some individuals inherited the putatively pathogenic variant from an unaffected parent points to reduced penetrance and a more complex inheritance, a phenomenon that can also not be excluded in the index family. Taken together, our data suggest that rare *KDR* variants may lead to TOF in the context of different inheritance paradigms that likely vary from monogenic to complex (oligogenic or polygenic).

We provide strong evidence that PTVs in *KDR* contribute to the pathogenesis of TOF. PTVs were approximately 45 times more prevalent among TOF cases compared to controls. In total we identified 11 PTVs in *KDR* in patients with TOF, not overlapping the two previously reported PTVs in patients with TOF.^10^ Although 8 of the 11 identified PTVs (73%) were located in the intracellular domain, these numbers are too small to make any statement about nonrandom distribution in TOF cases. Interestingly, we did not find any PTVs among a set of 2,312 patients with CHDs other than TOF, which may suggest that loss of function of this gene specifically predisposes to TOF within the spectrum of CHD. Of note, PTVs in *KDR* were recently also reported in patients with pulmonary arterial hypertension (PAH),^21,22^ although none of the PAH cases were reported to have CHD. What determines why some PTVs lead to TOF, while others might cause PAH, remains unclear.

Though not statistically significant, we observed a higher proportion of rare *KDR* missense variants in the extracellular domain of VEGFR2 in patients with TOF. One of these variants, *KDR-*p.(Val219Ala), was found in multiple patients and showed a marked reduced effect on VEGFR2 phosphorylation. This was in line with the *KDR* variants of the index family, and of a patient with TOF and absent pulmonary valve syndrome (*KDR-*p.([Thr442Met]), that were all located within the extracellular domain and also exhibited significantly reduced VEGFR2 phosphorylation. Similarly, missense variants in *FLT4*, encoding for VEGFR3, previously reported in patients with TOF, were primarily located in the extracellular domain.^8,10^ Clearly, larger studies are needed to confirm whether the extracellular domain is indeed specifically enriched in rare missense variants, as well as to explore differences in phenotype based on variant location in the protein. In addition, future studies should also assess whether there are phenotypic differences between patients with amorphic versus hypomorphic *KDR* variants.

The importance of VEGFR2 in heart development has long been established. While *Kdr* KO mice die early during development, conditional deletion of mouse *Kdr* in the endothelium (using an endothelial-specific Cre driver) causes embryonic lethality at E9.5–E10.5 associated with cardiac defects, including hypoplasia of the outflow tract (OFT) and the right ventricle, and loss of the endocardium,^23^ highlighting the role of VEGFR2 in OFT development. The second heart field (SHF) is essential for formation of the OFT and right ventricle,^24^ both regions of the heart affected in TOF. Conditional deletion of *Kdr* in the *Isl1* lineages, which includes the SHF, while showing preserved endocardium, leads to embryonic lethality at E14.5,^23^ supporting an important role of VEGFR2 in cardiogenesis independent from its role in the endocardium. The role of VEGFR2 in the SHF is further highlighted by the presence of Vegfr2 expression in the pharyngeal mesoderm at E8.5 and, at later stages, in (parts) of the SHF.^25^ Moreover, it was shown that the Tbx1 transcription factor, essential during OFT development, affects Vegfr2 expression in the SHF in vivo in mouse embryos and that *TBX1* favors a cardiac fate in VEGFR2 expressing cells.^25^ Given the above, we hypothesize that altered VEGFR2 expression and/or function within the SHF could directly or indirectly contribute to malformation of the OFT, and therefore TOF.

In an effort to provide evidence for causality of missense variants in *KDR* and determine their mechanism of pathogenicity, we engineered knock-in mouse lines harboring orthologues of the two variants identified in the index family. Contrary to the index family, where the disease phenotype was presumed to result from biallelic inheritance, compound heterozygous knock-in mice (i.e., *Kdr*^*G347W/G535R*^) were phenotypically normal. The lack of phenotype in the double heterozygous knock-in mice could be due to genetic background effects, a phenomenon that is firmly established for CHD.^26^ Although *Kdr*^*G347W/G347W*^ exhibited no morphological abnormalities, mice homozygous for the orthologous variant to the familial *KDR-*p.(Gly537Arg) variant (*Kdr*^*G535R/G535R*^) died during embryonic development and recapitulate the phenotype of constitutive *Kdr* knockout mice,^16^ underscoring the causality and severity of this variant. The varying severity of these two variants in mice in the homozygous state is reflected by the observed differences in severity in their Tyr1175 (p-VEGFR2) phosphorylation defect. Similar to the phosphorylation data in the mouse, in HEK 293T cells we observed a marked reduction in p-VEGFR2 in cells co-expressing the two variants or expressing *KDR*-p.(Gly537Arg) alone, while a milder decrease was seen in cells expressing the *KDR-*p.(Gly345Trp) variant. In aggregate, these data and the co-segregation data in the family support the concept that both alleles may be necessary for the development of the phenotype in the family, but contribute differentially to disease susceptibility, with the *KDR-*p.(Gly537Arg) variant having a larger effect than the *KDR-*p.(Gly345Trp) variant.

The extracellular domain of VEGFR2 is a critical part of the protein as it harbors the VEGF binding domain, and initiates receptor dimerization upon ligand binding.^11^ Furthermore, homotypic receptor–receptor contacts between Ig-like domains 4 and 7 further stabilize these VEGFR2 dimers and are essential for the exact positioning of the intracellular kinase domains,^27,28^ which institute protein kinase activation, trans-autophosphorylation (among others on Tyr1175), and initiation of signaling pathways. We suggest that the tested missense variants in the extracellular domain, i.e., *KDR*-p.(Gly345Trp), *KDR*-p.(Gly537Arg), *KDR-*p.(Val219Ala), and *KDR-*p.(Thr442Met), disturb this sequence of events, likely leading to diminished autophosphorylation and receptor activation. At the same time, in normal situations, ligand induced VEGFR2 activation stimulates the recycling of the intracellular VEGFR2 pool,^29^ as well as exit of newly synthesized VEGFR2 from the Golgi,^30^ thereby increasing the fraction of VEGFR2 on the plasma membrane. The intracellular accumulation of VEGFR2 that we observed for the *KDR*-p.(Gly537Arg) variant could indicate that this variant may interfere with this process. In turn, as receptors in intracellular vesicles are not accessible for VEGF, abnormal accumulation might further reduce the VEGFR2 phosphorylation levels. Regarding the *KDR* PTVs, we expect most if not all PTVs detected in TOF patients to result in nonsense-mediated decay (based on their location) and haploinsufficiency. We hypothesize that such reduced levels of VEGFR2 will impact on the total absolute amount of autophosphorylated VEGFR2, ultimately affecting proper function and development. Indeed phosphorylation of VEGFR2 at Tyr1175 has been shown to be essential for endothelial and hematopoietic development during embryogenesis.^31^ Yet, despite our finding of reduced autophosphorylation as a consequence of genetic variation in KDR, the exact involvement of reduced autophosphorylation in pathogenesis of TOF remains to be studied. While VEGFR2 signaling is extremely complex, and more than half a dozen pathways are recognized,^19^ a possible link between malfunctioning VEGFR2 phosphorylation and CHD comes from the knowledge that Tyr1175 phosphorylation is important in activation of the PLCϒ-ERK1/2 pathway^17,18^ and that disturbed ERK1/2 signaling contributes to cardiac defects that comprise TOF in vivo.^32,33^ Future studies are needed to address the exact downstream pathways affected by decreased VEGFR2 Tyr1175 phosphorylation.

### Limitations

Although we showed that the *KDR* variants in the index family are absent from publicly available reference data sets and 390 internal Moroccan controls, larger region-specific population genetic data sets will be required to fully confirm the rarity of the variants detected in Moroccan patients. In this study we only focused on rare variants in *KDR* and did not explore a multigenic inheritance. We did not functionally test all variants identified in patients and are therefore not able to conclude on their pathogenicity. We used individuals from the gnomAD collection as a control data set in the burden analysis. Although there are some inevitable limitations to this approach, it has been shown to be successful in other inherited cardiac disorders.^34^

### Conclusion

In conclusion, our data supports a role for rare *KDR* variants in pathogenesis of TOF through a loss-of-function mechanism. The total yield of rare *KDR* (VEGFR2) variants in TOF patients in this study (2.3%) is comparable to the previously reported yield of rare *FLT4* (VEGFR3) variants in patients with TOF. Taken together, the findings in this study shed light on the role of VEGF signaling in TOF and justify consideration of *KDR* screening in TOF patients in a clinical diagnostic setting.

## Supplementary information

Supplementary Methods, Tables 1-8, 11,Figure 1

Supplementary Tables 9-10,12-14

## Data Availability

Available upon request.
