# Correction to: Rare variants in *KDR*, encoding VEGF Receptor 2, are associated with tetralogy of Fallot

**DOI:** 10.1038/s41436-021-01279-7

**Published:** 2021-09-14

**Authors:** Doris Škorić-Milosavljević, Najim Lahrouchi, Fernanda M. Bosada, Gregor Dombrowsky, Simon G. Williams, Robert Lesurf, Fleur V. Y. Tjong, Roddy Walsh, Ihssane El Bouchikhi, Jeroen Breckpot, Enrique Audain, Aho Ilgun, Leander Beekman, Ilham Ratbi, Alanna Strong, Maximilian Muenke, Solveig Heide, Alison M. Muir, Mariam Hababa, Laura Cross, Dihong Zhou, Tomi Pastinen, German Competence Network for Congenital Heart Defects, Marc-Phillip Hitz, Hashim Abdul-Khaliq, Felix Berger, Ingo Dähnert, Sven Dittrich, Anselm Uebing, Brigitte Stiller, Elaine Zackai, Samir Atmani, Karim Ouldim, Najlae Adadi, Katharina Steindl, Anita Rauch, David Brook, Anna Wilsdon, Irene Kuipers, Nico A. Blom, Barbara J. Mulder, Heather C. Mefford, Boris Keren, Pascal Joset, Paul Kruszka, Isabelle Thiffault, Sarah E. Sheppard, Amy Roberts, Elisabeth M. Lodder, Bernard D. Keavney, Sally-Ann B. Clur, Seema Mital, Marc-Philip Hitz, Vincent M. Christoffels, Alex V. Postma, Connie R. Bezzina

**Affiliations:** 1 Department of Clinical and Experimental Cardiology, Amsterdam University Medical Center, Amsterdam, The Netherlands; 2 Department of Medical Biology, Amsterdam University Medical Center, Amsterdam, The Netherlands; 3 Department of Congenital Heart Disease and Pediatric Cardiology, Universitätsklinikum Schleswig-Holstein Kiel, Kiel, Germany; 4 Division of Cardiovascular Sciences, School of Medical Sciences, Faculty of Biology, Medicine and Health, The University of Manchester, Manchester, UK; 5 Genetics and Genome Biology Program, The Hospital for Sick Children, Toronto ON, Canada; 6 Laboratory of Medical Genetics and Oncogenetics, HASSAN II University Hospital, Fez, Morocco; 7 Center for Human Genetics Leuven and Catholic University Leuven, Leuven, Belgium; 8 Centre de Recherche en Génomique des Pathologies Humaines (GENOPATH), Faculté de Médecine et de Pharmacie, Mohammed V University of Rabat, Rabat, Morocco; 9 Département de génétique médicale, Institut National d’Hygiène, Rabat, Morocco; 10 Division of Human Genetics, Children’s Hospital of Philadelphia, Philadelphia, PA USA; 11 Center for Applied Genomics, Children’s Hospital of Philadelphia, Philadelphia, PA USA; 12 Medical Genetics Branch, National Human Genome Research Institute, National Institutes of Health, Bethesda, MD USA; 13 Département de génétique, Hôpital Pitié-Salpêtrière, APHP Sorbonne Université, Paris, France; 14 Department of Pediatrics, Division of Genetic Medicine, University of Washington, Seattle, WA USA; 15 Division of Clinical Genetics, Children’s Mercy Hospital, Kansas City, MO USA; 16 Center for Pediatric Genomic Medicine, Children’s Mercy Hospital and School of Medicine, University of Missouri–Kansas City, Kansas City, MO USA; 17 HASSAN II University Hospital, Fez, Morocco; 18 University of Sidi Mohammed Ben Abdellah, Fez, Morocco; 19 Faculty of Medicine and Pharmacy, Medical Genetics and Oncogenetics Unit, Sidi Mohamed Ben Abdellah University, Fez, Morocco; 20 Institute of Medical Genetics, University of Zurich, Zurich, Switzerland; 21 University of Nottingham, Queen’s Medical Centre, Nottingham, UK; 22 Department of Pediatric Cardiology, Amsterdam University Medical Center, Amsterdam, The Netherlands; 23 Department of Pediatric Cardiology, Leiden University Medical Center, Leiden, The Netherlands; 24 Department of Cardiology, Boston Children’s Hospital, and Department of Pediatrics, Harvard Medical School, Boston MA, USA; 25 Department of Clinical Genetics, Amsterdam University Medical Center, Amsterdam, The Netherlands; 26 Manchester University NHS Foundation Trust, Manchester Academic Health Science Centre, Manchester, UK; 27 The Hospital for Sick Children, Toronto ON, Canada; 28 University of Toronto, Toronto ON, Canada; 29 DZHK (German Centre for Cardiovascular Research) Partner Site, Kiel, Germany; 30 Wellcome Sanger Institute, Wellcome Genome Campus, Hinxton, Cambridge, UK; 31 Universitätsklinikum Schleswig-Holstein Kiel, Kiel, Germany; 32 Universitätsklinikum des Saarlandes, Homburg, Germany; 33 Deutsches Herzzentrum Berlin, Berlin, Germany; 34 Herzzentrum Leipzig, Leipzig, Germany; 35 Friedrich-Alexander-Universität Erlangen-Nürnberg, Erlangen, Germany; 36 Universitätsklinikum Schleswig-Holstein, Kiel, Germany; 37 Universitäts-Herzzentrum Freiburg Bad Krozingen, Freiburg, Germany

Correction to: *Genetics in Medicine* 2021; 10.1038/s41436-021-01212-y; published online 10 June 2021

Due to a processing error the author’s Doris Škorić-Milosavljević, Najim Lahrouchi, Alex V. Postma, Connie R. Bezzina were assigned to affiliation 38. However, affiliation 38 does not exist.

In addition, the affiliations of Najim Lahrouchi, Elisabeth M. Lodder, and Connie R. Bezzina should be number 1 instead of number 2. The correct affiliation is Department of Clinical and Experimental Cardiology, Amsterdam University Medical Center, Amsterdam, The Netherlands.

The original article has been corrected.

