# Supplementary material for: Rare variants in KDR, encoding VEGF Receptor 2, are associated with tetralogy of Fallot
Source: Genet Med. 2021 Jun 10;23(10):1952–60. doi: 10.1038/s41436-021-01212-y (PMC8486653; doi:10.1038/s41436-021-01212-y)
Supplement: Supplementary file 1 — Supplementary Methods, Tables 1-8, 11,Figure 1 [file 41436_2021_1212_MOESM1_ESM.pdf]

Online Supplemental Data

Rare variants in KDR, encoding VEGF receptor 2, are associated with tetralogy of Fallot

Content

Supplemental Methods 2

Supplementary Tables 1-8, 11 11

Supplementary Figure 1 20

References 21

Supplemental Methods

Exome sequencing index family

Written informed consent for clinical evaluation and genetic studies was obtained from all studied family members. Exome sequencing was performed on DNA isolated from peripheral blood leucocytes of the two affected children with tetralogy of Fallot (TOF) at the Beijing Genomics Institute (Shenzhen, China). Exons were captured using the Agilent Sureselect and sequenced on the HiSeq 2000 platform according to standard procedures. Sequence reads where then aligned to the reference sequence (NCBI build 37/hg19) using Burrows-Wheeler Aligner (BWA)[1]. Variant calls were made using SOAPsnp[2] for single nucleotide variants and SAMtools[3]/GATK[4] for small Insertion and deletions (InDels). We applied left-normalization to the InDels before annotating all variants using Annovar[5]. Only non-synonymous variants and variants at essential splice sites (≤ 2 bases from the 3' or 5' end of an intron) were considered. We combined the following variant reference databases: Exome Aggregation Consortium[6] (EXaC), Genome Aggregation Database[7] (gnomAD) (version 2.1.1), 1000 Genomes Project (1000GP)[8], 69 Genomes Data[9] (cg69) and The Greater Middle East (GME) Variome Project[10] and determined the highest reported minor allele frequency (MAF) of each variant (maxAF). We filtered out variants with maxAF >0.1%. Genes with multiple rare variants (2 or 3) were considered potentially compound heterozygous. Because of an underrepresentation of Moroccan samples in large-scale publicly available databases, we screened for the presence of the identified *KDR* variants in an internal set of 390 control individuals of Moroccan descent. Validation and co-segregation analysis in the two affected children, both parents and one healthy sibling were done using Sanger sequencing according to standard procedure. Primers are provided in **Supplemental Table 13.**

TaqMan assay in internal set of Moroccan controls

Using a TaqMan assay, we tested the variants *KDR*-p.(Gly347Trp) and *KDR*-p.(Gly535Arg) in an internal set of 390 healthy controls of Moroccan descent that were recruited in the Netherlands and in Morocco. The TaqMan reaction mixture consisted of 2,5µl of LightCycler 480 Probes Master

(Roche, no.: 4707494001) with 0.125µl of Taqman-probe-primer-mix (HEX-probe, recognizing the wildtype allele and FAM-probe, recognizing the mutant allele) and 1.375µl of mQ. Added to this was 1µl of 5-30ng/µl of genomic DNA. Next, the different alleles were amplified on the Light Cycler 480 -II machine (Roche) as follows: 10 min at 95 °C, followed by 40 cycles of a two-step amplification reaction consisting of: 15 seconds of 92 °C and 60 seconds at 60 °C. The last step consisted of 2 minutes cooling at 37 °C. After the program an endpoint genotyping analysis was performed to score the alleles.

Knock-in mouse production and phenotyping

Mouse lines harboring variants orthologues to the two rare KDR variants identified in the index family (*Kdr*-p.(Gly347Trp) and *Kdr*-p.(Gly535Arg)) were generated by CRISPR/Cas9 targeting at Cyagen (Santa Clara, CA 95050-2709, US). Cas9 mRNA, guide RNA and oligo donors harboring *Kdr*-p.(Gly347Trp) or *Kdr*-p.(Gly535Arg) were co-injected into fertilized eggs of mice of the FVB strain. Genotyping for the targeted variants was done by Sanger sequence analysis. Potential off-target modification at 5 loci with the highest affinity for the guide RNA were excluded by Sanger sequencing. The sequences of the guide RNA, the targeting oligo and the off-target loci analyzed are provided in **Supplementary table 14**. Interbreeding mice that carried one of the two different variants, i.e. *Kdr*-p.(Gly347Trp) or *Kdr*-(p.Gly535Arg), with each other or with wild-type mice resulted in the following knock-in mouse lines: mice heterozygous and homozygous for each of the variants separately ($Kdr^{G347W/+}$, $Kdr^{G347W/G347W}$, $Kdr^{G535R/+}$ *and* $Kdr^{G535R/G535R}$) and compound heterozygous mice carrying the two variants on separate alleles ($Kdr^{G347W/G535R}$).

Embryos were collected from timed matings by daily monitoring for vaginal mucus plugs. Occasional embryonic age corrections were made based on the number of somites of control littermate embryos.

We generated 5-8 litters of each relevant cross for Mendelian ratio testing of adults and neonates. For embryonic stages, we used 3-4 obtained 3-4 litters for each relevant cross. Both male and female mice were studied.

Animal care and experiments were in accordance with guidelines from the European Union, the Dutch government and the Amsterdam University Medical Center (UMC) and were approved by the Animal Experimental Committee of the Amsterdam UMC.

Whole mount images were obtained using a Leica MZ75 stereomicroscope. Hematoxylin and eosin (H&E) staining was performed on paraffin sections of the heart using standard methodology and staining reagents. Brightfield images of stained sections were collected using a Leica DM5000B widefield microscope.

Immunofluorescence staining

E10.0 *wild-type*, $Kdr^{G535R/+}$ and $Kdr^{G535R/G535R}$ mice embryos were fixed overnight in 4% paraformaldehyde in PBS, dehydrated in a graded ethanol series, paraffin embedded and sectioned at 7 µm. Sections were deparaffinized with xylenes and rehydrated in graded ethanol series. Sections were washed in PBS and pressure cooked for 5 minutes in antigen unmasking solution (Vector H-3300). Sections were blocked for 30 minutes in PBS-4% BSA followed by an overnight incubation with the primary antibody rabbit-anti-VEGFR2 (Cell signaling, CS, 55B11, 2479S) diluted 1:200 in PBS-4% BSA. Sections were washed and incubated with the secondary antibody Alexa Fluor 488 donkey-anti-rabbit IgG (1:200; Invitrogen, R37118) together with Alexa fluor 555 conjugate (1:500; Invitrogen, W32464) for 2 hours. Afterwards, sections were washed mounted in glycerol/PBS 1:1 and nuclei were stained with TO-PRO®-3 (1:1000; Invitrogen, T3605). Sections of the hearts were examined and confocal images were obtained on a Leica TCS SP8 DLS confocal mounted on a Leica DMi8 inverted microscope using an sCMOS camera.

No randomization was used. No blinding was done.

Phosphorylation assay

HEK 293T cells transfection

The full-length human *KDR* cDNA (pcDNA3.1+) was obtained from M. Flugelman (Leuven). The two variants of the index family, *KDR*-p.(Gly345Trp) and *KDR*-p.(Gly537Arg), as well as two additional variants identified in patients with TOF, *KDR*-p.(Val219Ala) and *KDR*-p.(Thr442Met), were introduced into the full-length human *KDR* construct by PCR-based site-directed mutagenesis (Q5 Site-Directed Mutagenesis Kit, NEB) and verified by sequencing and restriction enzyme digestion. HEK 293T cells were cultured in medium supplemented with 10% FBS, Penicillin-Streptomycin (100 U/ml) and 2 mM glutamine in a humidified incubator at 37 °C with 5% CO2. HEK 293T cells were transfected with constructs encoding (1) wild-type VEGFR2, (2) any of the four variants *KDR*-p.(Gly345Trp), *KDR*-p.(Gly537Arg) variant, *KDR*-p.(Val219Ala) or *KDR*-p.(Thr442Met) (with and without wild-type VEGFR2) or (3) the two variants from the index family, *KDR*-p.(Gly345Trp) and *KDR*-p.(Gly537Arg), together. Transfections were done as previously described by Van Engelen et al.[11]. Activation of VEGFR2 signaling was achieved by stimulating serum-starved cells with VEGF165 for 10 minutes as previously described[12]. Next, cells were washed with ice-cold scrape-solution and collected herein (500 µl/well) by scraping.

VEGF-165 Treatment Transfected HEK 239T Cells

Vascular Endothelial Growth Factor VEGF-165 (R&D SYSTEMS, 293-VE-010) was reconstituted at 100 ng/µl as a stock solution under sterile conditions in cell culture grade PBS containing 300 µg cell culture grade BSA, aliquoted and then stored at -80˚C. 40h post-transfection, cells were washed once with 1X PBS before being subjected to serum free culture medium (1 ml/well) for 2 hours. Thereafter, cells were subjected to 50 pg/µl VEGF-165 for 10 minutes. After VEGF-165 treatment for 10 minutes the cells were washed with ice-cold scrape-solution (2 mM PMSF, 2 mM EDTA, 2 mM EGTA, 2 mM NaF and 2 mM SodiumOrthovanadate in PBS 1X) and collected herein (500 µl/well) by scraping

Preparing Cell Lysates for protein quantification

Transfected HEK 239T Cells

Tubes containing VEGF-165 treated HEK 239T cells were spun in a bench top centrifuge at 4 °C, initially at 150xg for 3 minutes, subsequently at max speed (21000g) for 30 seconds. The supernatant was discarded and the pellets for protein isolation/extraction were suspended in 150 µl lysis solution (1% NP-40, 0.5% Sodium Deoxycholate, 0.1% SDS, 50 mM NaF, 2 mM Sodium Orthovanadate, 2 mM PMSF, 1 mM TCEP, 2 mM EGTA, 2 mM EDTA, 10 mM B-Glycerophosphate) supplemented with PhosStop (Roche) Inhibitor cocktail. Each collection was further lysed by 3 cycles of snap freezing in liquid Nitrogen, thawing on ice, homogenizing/lysing by gently pipetting up and down and incubating on ice for 10-15 minutes. After this, collections were centrifuged at maximum speed for 15 minutes at 4°C. The clarified cell lysate was transferred to clean micro centrifuge tubes. Cells from at least three independent transfections were pooled and homogenized by gentle pipetting. Protein quantification was done using the BCA Protein Assay (Thermo Scientific, 23227) with protein concentration measured on a NanoDrop 1000 (Thermo Scientific). Lysates were stored at -80 °C.

Yolk Sac cells from Knock-in Mice

Yolk sacs were isolated from E9.5-10.5 mouse embryos of wild-type mice and the different knock-in mice (i.e. $Kdr^{G347W/+}$, $Kdr^{G347W/G347W}$, $Kdr^{G535R/+}$, $Kdr^{G535R/G535R}$ and $Kdr^{G347W/G535R}$) and snap frozen in liquid nitrogen. These samples were lysed similar to described above, except 50 or 75 µl of lysis solution per tissue was used depending on sample size and 5 cycles of snap freezing.

Western Blot analysis

For each sample, 100 µg or 5-30 µg total protein (for HEK 293T lysates and yolk sac lysates respectively), was boiled in leamli sample solution in a final volume of 30 µl for 5 min at 95°C. Subsequently proteins were separated by SDS-PAGE (10% gel) and transferred to the blot (PVDF membrane, Immobilon-P, Millipore) using the semidry blotting technique. Subsequently the blot was blocked 2-3 hours at 4 °C in 5% BSA (Sigma, A7906) in 1X TBST (50 mM Tris, 150 mM NaCl, 0.1 % Tween-20) and incubated in this BSA solution overnight at 4 °C with the primary antibodies: either Rabbit Anti-VEGFR2 (CS, 55B11, 2479S, 1:500), Rabbit Anti-P-VEGFR2 (against Tyr1175, Thermo,

MA5-15170, 1:500) or Mouse Anti-B-Actin (Sigma, A5441, 1:5000). The next day the blot was washed 3 times quickly, and 3 times for ~20 minutes, with 1X TBST before a secondary antibody, comprising of Donkey Anti-Rabbit-HRP (GE Healthcare, NA930V, 1:5000) or Sheep Anti-Mouse-HRP (GE Healthcare, NA9310V, 1:5000), was added in 1% Protifar (Nutricia) milkpowder in 1X TBST for ~2 hours. Subsequently the blot was washed 3 times quickly, and 3 times every ~20 min, with 1X TBST prior to detection using ECL Select (GE Healthcare) for 5 minutes according to the manufacturer's procedure. The blots were imaged using the ImageQuant™ LAS 4000 imager and Western blot band intensity was quantified using ImageJ software (NIH v1.53a). The intensity of the VEGFR2 and phospho-VEGFR2 (p-VEGFR2) bands was normalized based on the beta-actin band. A total of 3-5 Western blots, from independent transfections, were performed and quantified. Values were factor corrected[15], combined, averaged and compared to wild-type values by two-sided t-test per condition. A p-value < 0.05 was considered significant.

Mutational analysis of *KDR* in selected set of patients with TOF

Patients included in this mutational analysis were derived from the Dutch national biobank of adult patients with CHD (CONCOR) or recruited at the Amsterdam UMC (Netherlands), at the Boston Children's hospital (United States) or at the University Hospital of Fez (Morocco).

The primers that were used in the polymerase chain reaction (PCR) to amplify the thirty exons and flanking intronic regions of *KDR* are provided in **Supplemental Table 13**. Sanger sequencing of the obtained PCR products was performed according to standard procedures to test for single nucleotide variants. To assess the presence of copy number variants we performed quantitative PCR (qPCR) with 9 ng of input DNA, which represented the amount of DNA of two copies of each loci. Moreover, 6 controls were included with a concentration of 3 ng, 9 ng and 27ng. Controls with a concentration of 3 and 27 ng represented the deletion and duplication amount respectively. Furthermore, each sample was measured three times in the same experiment. Five fluorescent probes were designed to target 5 loci across *KDR*, spread approximately 10 kb apart. Cycling was performed on a LC480 II PCR

Machine (Roche®) with the following settings for amplification, quantification and melting curve: Amplification: 95 °C 5 min, 95 °C 10 sec, 64 °C 10 sec, 72 ° 10 sec. Repeat step 2-4 45X. Quantification, integration time = 0.25 sec and integration time mode = manual . Melting Curve: 95 °C 5 sec, 65 °C 1 min, 95 °C continuous. In order to check specificity of amplification, melt curve analysis was performed using the LC480 II Melting Curve software and PCR products were checked on a 1.5% agarose gel. Subsequently, non-baseline corrected data were analyzed using LinRegPCR[13,14] and normalized against a reference amplicon.

Rare variants association analysis

Description of patient cohorts

Four different patient cohorts were combined for the rare variant association analysis. Two of which (the Toronto and UK cohorts) consisted of patients with isolated TOF, and two (the German cohort and the Pediatric Cardiac Genomics Consortium) consisted of patients with any type of CHD. Patients with a 22q11.2 deletion were excluded. Sample ancestry and relatedness was assessed in each cohort separately. They are described in more detail below.

Toronto cohort

The Toronto cohort consisted of isolated TOF probands recruited in the Ontario region in Canada and by the Dutch national biobank of adult patients with CHD (CONCOR). All samples were subjected to genome sequencing on the Illumina HiSeqX platform at The Center for Applied Genomics (Toronto). Alignment to the reference genome (hg19) was done using Isaac Aligner. Variants were called using Starling (Isaac Variant Caller) and were subsequently filtered to include only those with a 'PASS' flag in the 'FILTER' field. SNVs were additionally required to have a total filtered read depth ('DP') ≥ 10X, while indels were additionally required to have a total filtered read depth at the position preceding the indel ('DPI') ≥ 10X. Sample ancestry classifications were made using peddy (https://github.com/brentp/peddy). Relatedness was assessed using somalier (https://github.com/brentp/somalier).

UK cohort

This cohort refers to a previously published cohort of isolated TOF probands[15], recruited by researchers from the United Kingdom (UK). WES was performed using the Agilent SureSelectXT Human All Exon 50 Mb capture and libraries were sequenced on Illumina HiSeq2000. Details on downstream quality control and analysis are described elsewhere[15]. European cases were extracted in an identical way as described for the PCGC cohort. Relatedness was assessed using PLINK using SNP-array data on the same sample set[16].

German cohort

This cohort is a subset of a previously published cohort of cases with isolated CHD as well as CHD cases with extra-cardiac abnormalities[17]. Included CHD cases all had a severe and clinically relevant structural heart defect. Samples were sequenced on Illumina HiSeq after target enrichment using Agilent SureSelect V5 and V3. Quality control (QC) and filtering was performed using Hail 0.2. Samples and variants were considered for further analysis based on the following QC metrics and cut-offs: call rate >= 0.95, genotype quality average >= 25 and depth average >= 15. Genotypes (heterozygous) with allelic balance > 0.20 were retained in the analysis. The 1000GP Phase 3 dataset was used as reference population set for inferring European-matching sample ancestry. A Random Forest classifier was trained, using the first ten principal components (computed on a set of SNPs restricted to the Purcell interval), to predict the sample probability of belonging to one of five populations. The subset of European sample was defined as those individuals with a sample probability > 0.65 belonging to the European population.

PCGC cohort

The Pediatric Cardiac Genomics Consortium (PCGC) is a multi-center cohort including CHD cases from centers in the United States (US)[18]. All types of CHD were eligible in this study, with the exception of isolated patent foramen ovale, persistent ductus arteriosus related to prematurity, pulmonary stenosis related to twin-to-twin transfusion syndrome and isolated cardiomyopathy. CHD cases with missing phenotypic information (n=20) and those reported to have a recognized genetic syndrome (n=127) were excluded in the current study. Details on library preparation and sequencing have been

described previously[19]. Raw sequence data of all available CHD trios (n=2,648) was obtained through dbGaP (Study Accession: phs001194.v2.p2).

Alignment to the hg19 human reference genome was done using BWA. After InDel realignment and base recalibration using the Genome Analysis Tool Kit (GATK, version 4.1.4.1.) all samples were jointly genotyped producing GVCF files with the GATK haplotype caller. Variant quality filtering was done using GATK variant recalibrator and tranche values were set to 99.5 and 99.0 for SNPs and InDels, respectively. European ancestry was assessed with WES data following a previously published approach[20] and using the 1000GP as a reference dataset. Relatedness was assessed using PLINK.

Quality control steps

To account for the use of publicly available reference datasets we performed some quality control steps. This included a comparison of burden of synonymous variants, which are not expected to contribute to disease. Moreover, although the overall coverage of *KDR* was very good across gnomAD exomes, we accounted for any variable sequencing depth by adjusting to effective population size. Specifically, we took the *mean* allele number across all variants, per variant category, (divided by two) as the denominator. This reflects the proportion of individuals in whom these regions were adequately covered (i.e. with a "PASS" filter value), as opposed to the entire gnomAD population.

Filtering allele frequency

We used the population maximum filtering allele frequency (FAF) which is the highest true population allele frequency that is consistent with a variant's observed allele count[21], derived from allele frequencies from genome data of 71,702 individuals from gnomAD v3[7].

Supplementary Tables

Supplementary Table 1

VEGFR2 domains based on Roskoski[22]

Domain	Residues
All	1-1356
Ligand binding	141-320
Immunoglobulin-like 4-7	328-764
All extracellular	1-764
Protein kinase	834-1162
All intracellular	765-1356

Supplementary Table 2

Phenotype description affected siblings from the index family

Individual	II-1	II-3
**Main diagnosis**	TOF with absent pulmonary valve syndrome	TOF
**Overriding aorta**	>50% (double outlet right ventricle)	>50% (double outlet right ventricle)
**Pulmonary valve**	severly stenotic due to a small pulmonary valve annulus	severly hypoplastic
**Aortic arch**	left-sided	right-sided
**Ductus arteriosus**	Absent	Closed at birth (possible absent)
**Other**	persistent left superior vena cava	retro-aortic innominate vein and juxtaposition of the atrial appendages
**Age corrective surgery**	15 months	12 months

Supplementary Table 3

Genes previously implicated in TOF (n=77). Copied from Page et al. [15]

Gene name			
ALDH1A2	FOXC1	NHS	SEMA3D
ATP7A	FOXC2	NKX2.5	SEMA3E
BRAF	FOXH1	NKX2.6	SF3B4
BVES	GATA3	NODAL	SNX8
CACNA1C	GATA4	NOTCH1	STK11
CDKL5	GATA5	NOTCH2	STRA6
CFC1	GATA6	NRP1	TAB2
CHD4	GDF1	NR2F2	TBX1
CHD7	GJA5	PEX7	TBX20
CHUK	GPC5	PITX2	TBX5
CITED	HAND1	PLXNA2	TDGF1
COX7B	HAND2	PQBP1	TMEM260
CRKL	HEY2	PTPN11	TTC37
DDX11	HIBCH	RAB23	VEGFA
DDX59	HOXA1	RAD21	ZEB2
DISP1	JAG1	RAI1	ZFPM2/FOG2
DOCK6	KMT2D	RBM8A	ZIC3
EYA1	MESP1	ROCK1	
FANCL	MYOM2	RPL11	
FOXA2	NFATC	SALL4	

Supplementary Table 4

Results co-segregation analysis in index family

Gene	Chr	Pos	Ref	Alt	Protein	AF gnomAD v2	AF GME	CADD	Seggregation analysis
COL5A2	2	189914101	T	C	p.(Asn1040Ser)	2,40E-05	0	12	Paternal
COL5A2	2	189916186	G	A	p.(Pro931Ser)	0	0	23	Paternal
KDR	4	55972035	C	T	p.(Gly537Arg)	0	0	34	Maternal
KDR	4	55976879	C	A	p.(Gly345Trp)	0	0	35	Paternal

Abbreviations: AF, allele frequency; CADD, Combined Annotation Dependent Depletion; Chr, chromosome; GME, Greater Middle East Variome Project; gnomAD, The Genome Aggregation Database; Pos, position

Supplementary Table 5

Presence of *KDR* variants detected in index family in population genetics databases from North Africa and the Middle East. Databases were accessed on January 25th, 2021.

	Variant 1	Variant 2	Reference/website
**GRCh37**	chr4:g.55972035C>T	chr4:g.55976879C>A	
**GRCh38**	chr4:g.55105868C>T	chr4:g.55110712C>A	
**CDS**	c.1609G>A	c.1033G>T	
**Protein**	p.(Gly537Arg)	p.(Gly345Trp)	
**gnomAD v3 Middle East**	0/158	0/158	https://gnomad.broadinstitute.org
**GME Variome**	0/993	0/993	http://igm.ucsd.edu/gme/index.php
**EgyptRef**	0/109	0/109	https://www.egyptian-genome.org/
**Iranome**	0/800	0/800	http://www.iranome.ir
**Al mena (Qatar-1005)**	0/1,005	0/1,005	http://clingen.igib.res.in/almena/
**Total samples**	0/3,065	0/3,065	

Abbreviations: CDS, coding sequence; GME, Greater Middle East Variome Project; gnomAD, The Genome Aggregation Database; GRCh, Genome Reference Consortium Human genome build

Supplementary Table 6

Genotypes of pups from interbreeding *Kdr*$^{G347W/+}$ and *Kdr*$^{G347W/G535R}$ knock-in mice compared to expected Mendelian ratios (1:1:1:1)

Genotype	Expected	Actual
Kdr$^{G347W/G347W}$	14.25	13
Kdr$^{G347W/+}$	14.25	15
Kdr$^{G347W/G535R}$	14.25	16
Kdr$^{G535R/+}$	14.25	14
	χ2=0.345, df=3, P=0.951	

Supplementary Table 7

Genotypes of pups from interbreeding *Kdr*$^{G535R/+}$ knock-in mice and ratios compared to expected Mendelian ratios (1:2:1)

	At birth		E9.5		E10.5	
**Genotype**	**Expected**	**Actual**	**Expected**	**Actual**	**Expected**	**Actual**
Kdr$^{G535R/G535R}$	14,5	0	8,5	9	4,5	1*
Kdr$^{G535R/+}$	29	37	17	17	9	11
Kdr$^{+/+}$	14,5	21	8,5	8	4,5	6
Total	58		34		18	
	χ2=19.621; P=0.001		χ2=0.059; P=0.97		χ2=0.059; P=0.156	

**reabsorbed*

Supplementary Table 8

Characteristics of two *KDR* missense variants identified in index family

	p.(Gly345Trp)	p.(Gly537Arg)
gnomAD AF	0	0
Great Middle Eastern Variome AF	0	0
Moroccan controls AF	0	0
CADD	35	34
SIFT	Deleterious	Deleterious
PolyPhen	Probably damaging	Probably damaging
Location on protein	Ig-like domain 4	Ig-like domain 5
Orthologue in mouse	Gly347Trp	Gly535Arg
Knock-in mouse phenotype	Normal	Embryonically lethal in homozygous state with impaired endothelial development and enlarged pericardial cavity
p-VEGFR2 at Tyr1175	↓	↓↓
Subcellular localization	n/a	Intracellular accumulation

Abbreviations: AF, allele frequency; CADD, Combined Annotation Dependent Depletion; gnomAD, Genome Aggregation Database; n/a, not available

Supplementary Table 11

Number of patients per cohort for burden test analysis (after quality control) split by phenotype

Cohort	Toronto	UK[15]	German[17]	PCGC[19,23]	Total
**All TOF cases**	396	817	86	270	**1,569**
Isolated	396	817	62	177	**1,452**
With extra-cardiac abnormalities	0	0	24	93	**117**
**All CHD other than TOF**	n/a	n/a	923	1,389	**2,312**
Isolated	n/a	n/a	548	931	**1,479**
With extra-cardiac abnormalities	n/a	n/a	375	458	**833**

Abbreviations: CHD, congenital heart disease; PCGC, Pediatric Cardiac Genomics Consortium; TOF, Tetralogy of Fallot; UK, United Kingdom

Supplementary Figure

Supplementary Figure 1

Phosphorylation assay.

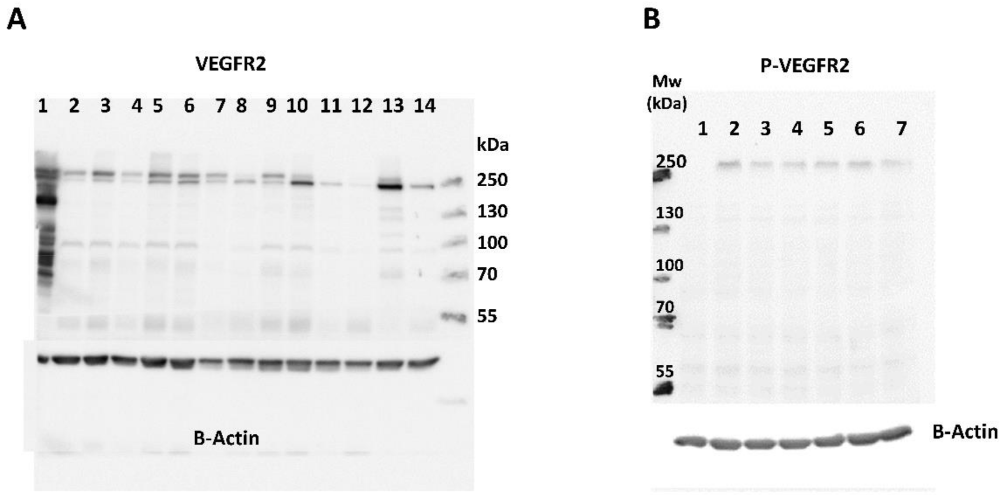

1	Wildtype
2	Wildtype
3	Wildtype
4	G347W Heterozygote
5	G347W Heterozygote
6	G347W Homozygote
7	Wildtype
8	G347W het/ G535R het
9	G347W Heterozygote
10	G347W het/ G535R het
11	G535R Homozygote
12	G535R Heterozygote
13	G535R Heterozygote
14	G535R Homozygote

		SF 2h	VEGF165 50 pg/µl,10 min	LOAD (µg)
1	pcDNA	+	+	100
2	WT	+	+	100
3	T446M	+	+	100
4	V219A	+	+	100
5	WT+T446M	+	+	100
6	WT+V219A	+	+	100
7	G345W+G537R	+	+	100

(A) Full unedited Western blot analysis with VEGFR2 antibody on yolk sac cells of wild-type and knock-in mice. Each lane represents one experiment. Genotypes are shown below in the table, this is the unedited version of figure 3C in the main text. Beta actin Western blot was imaged on top of VEGFR2 WB for easier quantification. **(B) Western blot analysis with p-Y1175 antibody in HEK 293T cells.** Representative example of P-VEGFR2 blots for the T446M and V219A variations. The lanes contain (from left to right) HEK293T cells transfected with an emtpy vector (pcDNA), wildtype *KDR* (WT), *KDR*-p.(Thr446Met) alone, *KDR*-p.(Val219Ala) alone, *KDR*-p.(Thr446Met) with WT, *KDR*-p.(Val219Ala) with WT and *KDR*-p.(Gly345Trp) together with *KDR*-p.(Gly537Arg). Beta actin Western blot was imaged on top of VEGFR2 WB for easier quantification.

References

1. Li, H. & Durbin, R. Fast and accurate short read alignment with Burrows-Wheeler transform. *Bioinformatics* **25**, 1754–1760 (2009).

2. Li, R., Li, Y., Fang, X., Yang, H., Wang, J., Kristiansen, K. & Wang, J. SNP detection for massively parallel whole-genome resequencing. *Genome Res.* **19**, 1124–1132 (2009).

3. Li, H., Handsaker, B., Wysoker, A., *et al.* The Sequence Alignment/Map format and SAMtools. *Bioinformatics* **25**, 2078–2079 (2009).

4. McKenna, A., Hanna, M., Banks, E., *et al.* The genome analysis toolkit: A MapReduce framework for analyzing next-generation DNA sequencing data. *Genome Res.* **20**, 1297–1303 (2010).

5. Wang, K., Li, M. & Hakonarson, H. ANNOVAR: functional annotation of genetic variants from high-throughput sequencing data. *Nucleic Acids Res.* **38**, e164 (2010).

6. Lek, M., Karczewski, K. J., Minikel, E. V, *et al.* Analysis of protein-coding genetic variation in 60,706 humans. *Nature* **536**, 285–91 (2016).

7. Karczewski, K. J., Francioli, L. C., Tiao, G., *et al.* The mutational constraint spectrum quantified from variation in 141,456 humans. *Nature* **581**, 434–443 (2020).

8. 1000 Genomes Project Consortium, R. A., Auton, A., Brooks, L. D., *et al.* A global reference for human genetic variation. *Nature* **526**, 68–74 (2015).

9. Drmanac, R., Sparks, A. B., Callow, M. J., *et al.* Human genome sequencing using unchained base reads on self-assembling DNA nanoarrays. *Science (80-.).* **327**, 78–81 (2010).

10. Scott, E. M., Halees, A., Itan, Y., *et al.* Characterization of greater middle eastern genetic variation for enhanced disease gene discovery. *Nat. Genet.* **48**, 1071–1079 (2016).

11. van Engelen, K., Mommersteeg, M. T. M., Baars, M. J. H., *et al.* The Ambiguous Role of NKX2-5 Mutations in Thyroid Dysgenesis. *PLoS One* **7**, e52685 (2012).

12. Antonescu, C. R., Yoshida, A., Guo, T., *et al.* KDR activating mutations in human angiosarcomas are sensitive to specific kinase inhibitors. *Cancer Res.* **69**, 7175–7179 (2009).

13. Ruijter, J. M., Ramakers, C., Hoogaars, W. M. H., Karlen, Y., Bakker, O., van den hoff, M. J. B. & Moorman, A. F. M. Amplification efficiency: Linking baseline and bias in the analysis of quantitative PCR data. *Nucleic Acids Res.* **37**, (2009).

14. Ramakers, C., Ruijter, J. M., Lekanne Deprez, R. H. & Moorman, A. F. M. Assumption-free analysis of quantitative real-time polymerase chain reaction (PCR) data. *Neurosci Lett.* **339**, 62–66 (2003).

15. Page, D. J., Miossec, M. J., Williams, S. G., *et al.* Whole Exome Sequencing Reveals the Major Genetic Contributors to Non-Syndromic Tetralogy of Fallot. *Circ. Res.* **124**, 553–563 (2018).

16. Cordell, H. J., Topf, A., Mamasoula, C., *et al.* Genome-wide association study identifies loci on 12q24 and 13q32 associated with Tetralogy of Fallot. *Hum. Mol. Genet.* **22**, 1473–1481 (2013).

17. Sifrim, A., Hitz, M.-P., Wilsdon, A., *et al.* Distinct genetic architectures for syndromic and nonsyndromic congenital heart defects identified by exome sequencing. *Nat. Genet.* **48**, 1060–1065 (2016).

18. Hoang, T. T., Goldmuntz, E., Roberts, A. E., *et al.* The Congenital Heart Disease Genetic Network Study: Cohort description. *PLoS One* **13**, e0191319 (2018).

19. Jin, S. C., Homsy, J., Zaidi, S., *et al.* Contribution of rare inherited and de novo variants in 2,871 congenital heart disease probands. *Nat. Genet.* **49**, 1593–1601 (2017).

20. Belkadi, A., Pedergnana, V., Cobat, A., *et al.* Whole-exome sequencing to analyze population structure, parental inbreeding, and familial linkage. *Proc. Natl. Acad. Sci. U. S. A.* **113**, 6713–6718 (2016).

21. Whiffin, N., Minikel, E., Walsh, R., *et al.* Using high-resolution variant frequencies to empower clinical genome interpretation. *Genet. Med.* **19**, 1151–1158 (2017).

22. Roskoski, R. VEGF receptor protein-tyrosine kinases: Structure and regulation. *Biochem. Biophys. Res. Commun.* **375**, 287–291 (2008).

23. Homsy, J., Zaidi, S., Shen, Y., *et al.* De novo mutations in congenital heart disease with neurodevelopmental and other congenital anomalies. *Science (80-.).* **350**, 1262–1266 (2015).
